# Structural Characteristics and Formation Mechanism of Microbiota Related to Fermentation Ability and Alcohol Production Ability in Nongxiang *Daqu*

**DOI:** 10.3390/foods11172602

**Published:** 2022-08-27

**Authors:** Jie Tang, Jie Chen, Deming Chen, Zijian Li, Dan Huang, Huibo Luo

**Affiliations:** 1 College of Bioengineering, Sichuan University of Science and Engineering, Zigong 643000, China; 2 Yibin Nanxi Wine Co., Ltd., Yibin 644000, China; 3 Brewing Biotechnology and Application Key Laboratory of Sichuan Province, Yibin 644000, China

**Keywords:** medium temperature *Daqu*, functional microbiota, community structure, microbial succession, correlation analysis

## Abstract

Fermentation ability and alcohol production ability are important quality indicators of Chinese liquor *Daqu*, reflecting microbial growth and metabolic capacity and ethanol production capacity of *Daqu* microbiota, respectively. However, information on the microbial community related to the fermentation ability and alcohol production ability is unclear. In this study, fermentation functional microbiota (FFM) and alcohol functional microbiota (AFM) were obtained by correlating fermentation ability and alcohol production ability with *Daqu* microbiota. FFM and AFM consisted of 50 and 49 genera, respectively, which were basically the same at the phylum level but differed at the genus level. Correlation analysis showed that FFM and AFM were mainly affected by moisture, acidity, and humidity in the early stage of *Daqu* fermentation, and oxygen content was a critical factor for microbial succession in the middle stage of fermentation. FFM and AFM had commensal or synergistic interactions with multiple microbes. Function predictions indicated that fermentation functional bacterial microbiota was active in product synthesis and transport-related metabolic functions, and alcohol functional bacterial microbiota was very active in raw material utilization and its own metabolic synthesis. This study reveals the structural characteristics and formation mechanism of FFM and AFM, which is important for control of *Daqu* quality.

## 1. Introduction

Chinese *Baijiu* is one of the six major distilled spirits in the world and is widely loved by consumers for its unique aroma and taste. *Daqu*, the saccharifying fermenting agent in the solid-state fermentation process of Chinese *Baijiu*, provides raw materials, microbes, and enzymes vital to *Baijiu* fermentation. While brewing *Baijiu*, *Daqu* is generally added after *Jiupei* (a mixture of fermented grains) has cooled, mixed well, and then fermented in the pit. During fermentation, *Daqu* is rich in hydrolytic enzymes and microflora, thus allowing the simultaneous saccharification and fermentation of grains. During *Baijiu* production, starch is hydrolyzed to glucose, which is further converted to ethanol. This process is continuous, progressive, and involves various microbes and enzymes. Liquefaction activity, saccharification activity, fermentation ability, and alcohol production ability are biochemical indicators reflecting *Daqu* quality. While liquefaction activity and saccharification activity are related to enzymes, fermentation ability and alcohol production ability are more concerned with microbes in *Daqu*.

Fermentation ability is expressed in terms of carbon dioxide produced by *Daqu* using fermentable sugars. Multiple metabolic pathways in microbial metabolism can produce carbon dioxide, accompanied by the production of a large number of intermediate metabolites that contribute to the formation of flavor compounds. *Daqu* with high fermentation ability also has a stronger aroma [1], and *Daqu* aroma is an important sensory indicator to determine whether *Daqu* is of a high quality. Moreover, high-quality *Daqu* often has higher fermentation ability than ordinary *Daqu* [2]. Thus, fermentation ability can explain *Daqu* aroma to some extent, indicating that it occupies an important place in evaluation *Daqu* quality. Alcohol production ability is expressed by the conversion of fermentable sugars to ethanol by *Daqu*, directly reflecting its liquor production ability, and is an important indicator of *Daqu* quality. In practice, alcohol production ability and fermentation ability are often used together to explain the liquor production ability of *Daqu*. However, microbial metabolism does not necessarily imply ethanol production in parallel with carbon dioxide production. We argue that alcohol production ability is a more direct representation of the ethanol production capacity of *Daqu* microbiota, and fermentation ability is a more comprehensive reflection of the growth and metabolic capacity of *Daqu* microbiota.

Essentially, the fermentation ability and alcohol production ability represent the metabolic activities performed by microbes associated with them in *Daqu*. Yeast is closely related to the fermentation ability and alcohol production ability. The amount of yeast in high-quality *Daqu* differs from ordinary *Daqu* by an order of magnitude while fermentation ability is the highest in high-quality *Daqu* [3]. However, *Daqu* is co-fermented by several species, and fermentation ability and alcohol production ability may be associated with various microbes. The fermentation ability of high-quality roasted sesame-flavored *Daqu* was significantly higher than that of ordinary *Daqu*, and this difference was due to differences in the corresponding microbiota [2]. Similarly, the fermentation ability of different color high-temperature *Daqu* was significantly different because of the diversity of *Daqu* microbes and metabolites [4]. The abundance of ethanol-related coding genes in *Daqu* significantly increased after *Bacillus* inoculation [5]. The ethanol content of *Daqu* increased after fortification with *Saccharomycopsis* and *Absidia* [6]. Such is the effect of fortified strains on the community structure and thus on the ethanol content. It can be seen that changes in fermentation ability and alcohol production ability are related to changes in microbial taxa. Despite reports of studies on the correlation between fermentation ability and alcohol production ability and microbes, an in-depth analysis of the associated microbial community structure is lacking.

According to ecological theory, when community structure and species composition are relatively stable, community influencing factors reach a dynamic balance, and the community is in sustainable development, marking the formation of the climax community [7,8]. During *Daqu* fermentation, the microbial community is regulated by many factors, and a stable climax community is basically formed at the end of fermentation. It is our concern how primary community of *Daqu* microbiota succession to climax community. The succession of *Daqu* communities is not only significantly related with temperature, moisture, humidity, and acidity but also regulated by microbial interactions [9,10,11]. Thus, the formation of the climax microbial community of *Daqu* might be influenced by both biotic and abiotic factors. However, little is known about the factors influencing the fermentation ability and alcohol production ability-related communities. It is important to reveal the structural characteristics and formation mechanism of the microbial communities related to the fermentation ability and alcohol production ability for the control of quality of *Daqu*.

In this study, the correlated microbial communities were obtained by correlating *Daqu* microbiota with the fermentation ability and alcohol production ability. The structural differences between the two correlated microbial communities were further analyzed. Correlation analysis was performed to study the effects of environmental factors and microbial interactions on the correlated communities to reveal community formation mechanisms. Function predictions analysis explored the potential functions of the two correlated microbial communities. Finally, this study can provide a theoretical basis for the production of standard quality *Daqu*.

## 2. Materials and Methods

### 2.1. Sampling

*Daqu* bricks were collected from a *Nongxiang Baijiu* enterprise in Yibin, China. The bricks were collected at different time points (days 1, 2, 5, 6, 12, and 13) during *Daqu* fermentation. A *Daqu* brick was taken from four locations of a Qu-room each time (Figure S1). Each *Daqu* brick was crushed and stored in a sterile plastic bag. A total of 24 samples were collected, and a part of them was analyzed for physicochemical properties in real time, while the other part was stored at −80 °C for total DNA extraction.

### 2.2. Physicochemical Properties

Total acidity was assessed using the acid base titration method. The moisture of *Daqu* was determined by the direct drying method at 105 °C. The temperature, humidity, and oxygen content at each sampling point of the *Daqu* bricks were monitored in real time using the sensors of an online environmental detection system. Fermentation ability and alcohol production ability were determined as follows: 1 g of *Daqu* powder was added to 150 mL of sterilized saccharification solution, which was placed in an incubator at 30 °C, and the fermentation was stopped after 72 h. Fermentation ability was evaluated by measuring the weight change of the fermented system before and after fermentation, i.e., the amount of carbon dioxide produced ((unit: g/(1 g-72 h); symbol: U). Alcohol production ability was expressed as the ethanol content in the fermentation broth at the end of fermentation (unit is mL/100 g). Because of the low ethanol content in some samples, a modified method of residual alcohol determination in *Jiupei* was used to determine the ethanol content in the samples [12]. Potassium dichromate oxidizes ethanol to acetic acid, and hexavalent chromium is reduced to trivalent chromium; thus, the ethanol content of the fermentation broth was determined by the colorimetric method. The method was as follows: 100 mL of fermentation solution was taken for distillation, an appropriate amount of distillate was taken in a colorimetric tube, 1 mL of 2% potassium dichromate and 5 mL of concentrated sulfuric acid were added. The solution was shaken well, heated together with the standard tube for 10 min, and cooled. Finally, the optical density was measured at 600 nm with a spectrophotometer.

### 2.3. DNA Extraction and Processing

To obtain the genomic information of the microbial community, the total DNA in the samples was extracted using a modified cetyltrimethylammonium bromide extraction method [13]. The V3–V4 region of the bacterial 16S rRNA gene was amplified using the universal primer pair 338F/806R, and the internal transcribed spacer (ITS) region of the fungal gene was amplified using the universal primer pair ITS1F/2043R. PCR amplification was performed as follows: 95 °C for 3 min; 95 °C for 30 s, 55 °C for 30 s, 72 °C for 45 s, 27 cycles; 72 °C for 10 min, ending at 4 °C. PCR products were separated on a 2% agarose gel, purified using the AxyPrep DNA gel extraction kit (Axygen Biosciences, Union City, CA, USA) and quantified using the Quantus™ fluorometer (Promega, Madison, WI, USA). Amplicons were sequenced using the Illumina MiSeq platform at Majorbio Bio-Pharm Technology Co., Ltd. (Shanghai, China). The raw sequence reads generated in this study were all deposited in the NCBI database (accession number: PRJNA869206).

### 2.4. Processing of Sequencing Data

In short, raw sequence data were demultiplexed and quality-filtered using fastp v0.19.6 (https://github.com/OpenGene/fastp) [14] and merged by FLASH v1.2.11 (https://ccb.jhu.edu/software/FLASH/index.shtml) [15]. Operational taxonomic units (OTUs) with 97% similarity cutoff [16,17] were clustered using UPARSE v11 (http://www.drive5.com/uparse/) [16], and chimeric sequences were identified and removed. The taxonomy of each OTU representative sequence was analyzed by RDP Classifier v2.13 (https://sourceforge.net/projects/rdp-classifier/) [18] against a 16S rRNA database (Silva v138) and an ITS rRNA database (Unite 8.0) using the confidence threshold of 0.7.

### 2.5. Statistical Analysis

Spearman correlations between the two indicators (i.e., fermentation ability and alcohol production ability) and *Daqu* microbiota were analyzed using the pheatmap package in R v3.3.1. Redundancy analysis (RDA) or canonical correspondence analysis (CCA) was performed to reveal the correlations between associated microbial communities and environmental factors using the vegan package in R. To reveal the effects of microbial interactions on the associated communities, Procrustes analysis and co-occurrence network analysis were performed using the vegan and igraph packages in R, respectively. Phylogenetic investigation of communities by reconstruction of unobserved states (PICRUSt2) analysis based on 16S rRNA and ITS sequence data was performed to gain additional insights into the metabolic potential of the related communities. The gene family counts for each predicted sample were derived from the MetaCyc metabolic pathway database (https://metacyc.org/, accessed on 5 June 2022). The differential functions of the two microbial communities were analyzed using linear discriminant analysis effect size (LEfSe; http://huttenhower.sph.harvard.edu/galaxy/ , accessed on 20 June 2022).

## 3. Results and Discussion

### 3.1. Variations in the Fermentation Ability and Alcohol Production Ability

Variations in the fermentation ability and alcohol production ability are shown in Figure S2. At the beginning of fermentation, fermentation ability was high, but it gradually decreased with an increase in temperature, eventually dropped to 0.22 (U). In the first two days of fermentation, alcohol production ability was low, and but it gradually increased to 3.03 (mL/100 g), the highest level, which lasted for 4 days, before rapidly decreasing and finally returning to the initial level. This might be due to microbial succession during fermentation, which leads to changes in fermentation ability and alcohol production ability.

### 3.2. Structural Characteristics of Functional Microbiota

**Figure 1:**
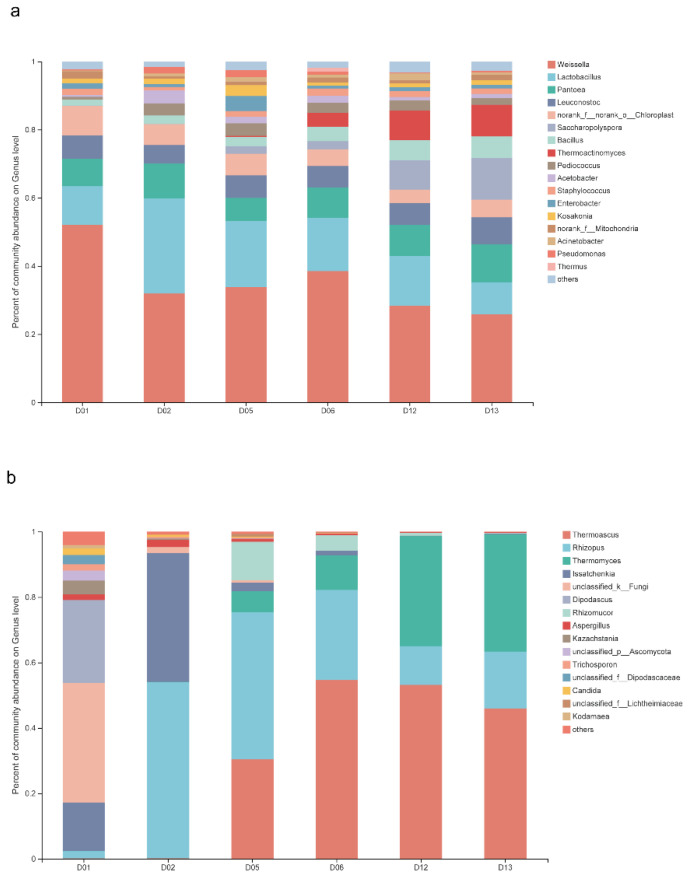
*Daqu* microbiota composition during fermentation. Bar plots of bacterial genera (**a**), fungal genera (**b**), and “others” include all taxa <0.01%.

High-throughput sequencing technology was used to obtain information on *Daqu* microbiota, in which there were 211 genera in 12 bacterial phyla and 63 genera in 4 fungal phyla. The community barplots in Figure 1 show the composition and dynamics of *Daqu* microbiota. The relative abundance (RA) of each microbial species in the community barplot was greater than 0.01%. The richness of bacterial genera increased with the increase in days of fermentation, but the community changes usually tended to be stable (Figure 1a). The abundance of fungal genera decreased with the increase in days of fermentation, and the rate of fungal succession was rapid (Figure 1b).

**Figure 2:**
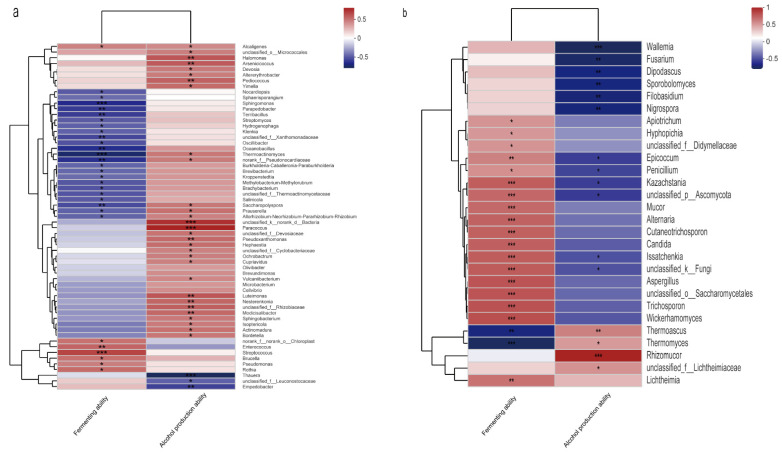
Heatmap of correlation analysis between *Daqu* microbiota and the fermentation ability and alcohol production ability. (**a**) Bacterial genera. (**b**) Fungal genera (|R| > 0.4, *p* < 0.05, * 0.01 < *p* ≤ 0.05, ** 0.001 < *p* ≤ 0.01, *** *p* ≤ 0.001).

To obtain the associated microbial communities, we performed correlation analysis of *Daqu* microbiota with fermentation ability and alcohol production ability. The microbial communities that showed significant correlation (|R| > 0.4, *p* < 0.05) with the two indicators were termed fermentation functional microbiota (FFM) and alcohol functional microbiota (AFM). The fermentation functional bacterial microbiota (FFBM) comprised 30 genera in 5 phyla—Cyanobacteria, Bacteroides, Proteobacteria, Firmicutes, and Actinobacteria. A total of 7 genera were positively correlated and 23 genera were negatively correlated with fermentation ability (Figure 2a). The alcohol functional bacterial microbiota (AFBM) consisted of 33 genera in 5 phyla—Bacteroides, unclassified_k__norank_d__Bacteria, Proteobacteria, Firmicutes, and Actinobacteria. Thirty genera were positively correlated and three genera were negatively correlated with alcohol production ability. At the phylum level, both had the same four phyla, but at the genus level, 24 genera were specific to FFBM, 27 genera were specific to AFBM, and only 6 genera were shared by FFBM and AFBM. In FFBM, the dominant genera were *norank_f__norank_o__Chloroplast*, *Saccharopolyspora*, *Thermoactinomyces*, and *Pseudomonas*. In AFBM, the dominant genera were *Saccharopolyspora*, *Thermoactinomyces*, and *Pediococcus*. In AFBM, *Pediococcus* (with RA reaching 3.5%) was the dominant genus on the first day and the absolute dominant genus on the second day of fermentation. In FFBM, *norank_f__norank_o__Chloroplast* (with RA reaching 52%) was the dominant genus on the first day of fermentation. RA of both subsequently decreased with fermentation time. *Pseudomonas* in FFBM is a dominant genus in the fermentation pre-period and significantly regulates bacterial diversity and community composition [19]. In addition, it regulates the spinability of dimethylbutyrate, which is an important flavor compound [20]. *Saccharopolyspora* and *Thermoactinomyces* were the dominant genera shared by the two communities. *Saccharopolyspora* is a key genus in the middle stage of *Daqu* fermentation [9] and may be involved in amino acid metabolism, sugar metabolism, and ester synthesis in *Daqu* fermentation [21]. *Thermoactinomyces* is a thermotolerant bacterial genus that is dominant in both *Daqu* and heap fermentation [22] with complete metabolic pathways for acetaldehyde and acetic, propionic, and butyric acids [23], which are crucial to the formation of volatile compounds [24].

Fermentation functional fungal microbiota (FFFM) comprised 20 genera in 4 phyla—Mucoromycota, unclassified_k__Fungi, Ascomycota, and Basidiomycota (Figure 2b). Eighteen genera were positively correlated with fermentation ability, of which yeasts accounted for one-half—*Cutaneotrichosporon*, *Kazachstania*, *Trichosporon*, *Candida*, *Wickerhamomyces*, *Issatchenkia*, *Apiotrichum*, *Hyphopichia*, and *unclassified_o__Saccharomycetales*, indicating that carbon dioxide production was closely related with yeasts, which is consistent with previous investigations [3]. Alcohol functional fungal microbiota (AFFM) included 16 genera in the same 4 phyla (i.e., Mucoromycota, unclassified_k__Fungi, Ascomycota, and Basidiomycota). A total of 4 genera were positively correlated and 12 genera (mainly molds and yeasts) were negatively correlated with alcohol production ability. Both FFFM and AFFM were identical at the phylum level, but at the genus level, 12 genera were exclusive to FFFM, 8 genera were exclusive to AFFM, and 8 genera were common to FFFM and AFFM. Dominant genera in FFFM were *Thermoascus*, *Thermomyces*, *Issatchenkia*, *unclassified_k__Fungi*, *Aspergillus*, *Kazachstania*, *Trichosporon*, *unclassified_p__Ascomycota*, and *Candida*. Dominant genera in AFFM were *Thermoascus*, *Thermomyces*, *Issatchenkia*, *unclassified_k__Fungi*, *Dipodascus*, *Rhizomucor*, *Kazachstania*, *unclassified_p__Ascomycota*, and *unclassified_f__ Lichtheimiaceae*. Six genera were common to both communities, three genera were unique to FFFM, and three genera were unique to AFFM. *Thermoascus*, *Thermomyces*, *Issatchenkia*, and *unclassified_k__Fungi* were the dominant genera shared by the two communities, and the sum of RA of these four genera was greater than 74% on each day of fermentation. *Thermoascus* and *Thermomyces* can be used as biomarkers for soy sauce flavor *Daqu* for black and white types, respectively [25], and are the dominant genera in different rounds of heap fermentation [26] as well as the dominant genera in the early stage of pit fermentation [27], which can produce various thermophilic glycoside hydrolases [28] to accelerate the decomposition and utilization of starchy raw materials, promote microbial growth, and produce *Baijiu* flavor [29]. *Aspergillus* is also a dominant genus in *Daqu* at the fermentation stage, heap stage, and early stage of pit fermentation [26,27,30], and it synthesizes various carbohydrate hydrolases that directly participate in the starch, pyruvate, and tricarboxylic acid cycle (TCA) pathways [31], laying the material foundation for producing liquor and aroma by other strains. *Kazachstania* is the dominant genus in the early stage of *Daqu* fermentation, and it can replace the dominant genera *Aspergillus*, *Thermoascus,* and *Thermomyces* as the absolute dominant genus in the middle and late stages of liquor fermentation [27]. It has some acid tolerance and can thus participate in the biosynthesis of acetic acid, lactic acid, ethyl acetate, and ethyl lactate [32,33], contributing greatly to the late stage of *Baijiu* aroma development. *Issatchenkia* is the absolute dominant genus in the first two days, with RA reaching 85% on the second day of fermentation. It synthesizes pectinases and lipases [34] that are associated with the production of higher alcohols and volatile acids in grain fermentation [22,35]. *Trichosporon* is a key genus in *Daqu* fermentation [9], and it is associated with phenylethanol synthesis in sauce-flavored *Daqu* [29], which has a rosy aroma and is an important *Baijiu* flavor compound. *Candida* is the dominant genus in different stages [26,27], and it produces various hydrolytic enzymes [36], which have the ability to synthesize acetic acid and many alcohols [33,37]. We can conclude that *Aspergillus*, *Thermoascus*, and *Thermomyces* in the fungal community were important fermentation starters in liquor fermentation, and these fungal genera could break down starch raw materials in the early stage of fermentation to form various small molecules that provide the material basis for the metabolic synthesis of the other strains. The four non-*Saccharomyces* yeast strains *Kazachstania*, *Issatchenkia*, *Trichosporon*, and *Candida* play an articulate role and can synthesize various flavor chemicals in the middle and late stages of *Baijiu* fermentation, contributing to the aroma of the spirit.

In terms of total genera, FFBM and AFBM accounted for nearly one-seventh and one-sixth of the total bacterial genera, respectively, and FFFM and AFFM accounted for nearly one-third and one-fourth of the total fungal genera, respectively. In terms of dominant genera, FFBM and AFBM contained 4 and 3 of the 17 dominant genera in the bacterial community, respectively, and FFFM and AFFM each contained 9 of the 15 dominant genera in the fungal community. Thus, FFM and AFM play pivotal roles in the whole *Daqu* community.

### 3.3. Effects of Biotic and Abiotic Factors on Functional Microbial Communities

**Figure 3:**
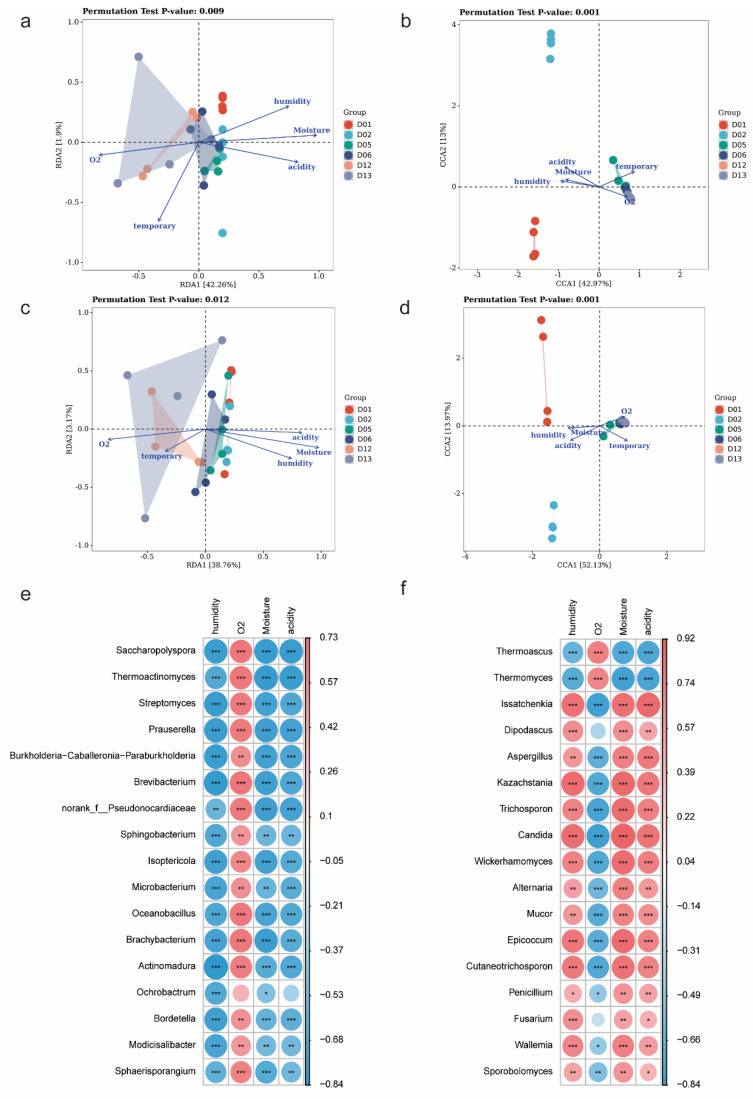
Effects of environmental factors on functional microbial communities. The effects of environmental factors on AFBM (**a**), AFFM (**b**), FFBM (**c**), and FFFM (**d**) were studied using RDA/CCA. The length of the arrow represents the size of the effect of environmental factors on the distribution of species in the sample. Heatmap of the association between environmental factors and functional microbiota. Bacterial genera (**e**), fungal genera (**f**). Red represents positive correlation, blue represents negative correlation, and the degree of correlation is proportional to the size of the circle. (* 0.01 < *p* ≤ 0.05, ** 0.001 < *p* ≤ 0.01, *** *p* ≤ 0.001).

To investigate the effects of environmental factors on the correlated communities, RDA or CCA was performed. As shown in Figure 3a–d, the two axes explained 44.15%, 55.96%, 41.92%, and 66.10% of the total variance of AFBM, AFFM, FFBM, and FFFM differentiation, respectively, indicating that the physicochemical properties could explain the variation in microbial communities to some extent. AFBM and FFBM were affected by moisture, acidity, and oxygen content to a significant level (Table S1) (r2 > 0.4, *p* < 0.01). Moisture, acidity, humidity, oxygen content, and temperature were significantly correlated with AFFM and FFFM distribution (r2 > 0.4, *p* < 0.01). Therefore, the effects of environmental factors on FFM and AFM succession were approximately the same, i.e., FFM and AFM were mainly affected by moisture, acidity, and humidity in the early stage of *Daqu* fermentation, and oxygen content became a critical factor for microbial succession in the middle stage of fermentation. Further, change in moisture was the main factor affecting succession in FFBM and AFBM (r2 ≥ 0.60, *p* < 0.01), which is in line with the fact that the main driving force of the *Daqu* bacterial community is moisture [11,38], and all factors significantly contributed to succession in FFFM and AFFM, with humidity having the most significant effect (r2 ≥ 0.84, *p* < 0.01).

As shown in Figure 3e,f, Spearman correlation analysis revealed that 16 bacterial genera and 15 fungal genera were significantly correlated with the 4 environmental factors (r2 > 0.6, *p* < 0.05). Bacterial genera of *Saccharopolyspora*, *Thermoactinomyces*, *Streptomyces*, *Prauserella*, *Burkholderia-Caballeronia-Paraburkholderia*, *Brevibacterium*, *norank_f__Pseudonocardiaceae*, *Sphingobacterium*, *Isoptericola*, *Microbacterium*, *Oceanobacillus*, *Brachybacterium*, *Actinomadura*, *Bordetella*, *Modicisalibacter*, and *Sphaerisporangium* were positively correlated with oxygen content (*p* < 0.01) and negatively correlated with the other three environmental factors (*p* < 0.05). Similarly, *Thermoascus* and *Thermomyces* in the fungal community showed a positive correlation with oxygen content (*p* < 0.001) and negative correlation with humidity, moisture, and acidity (*p* < 0.001). In contrast, *Issatchenkia*, *Aspergillus*, *Kazachstania*, *Trichosporon*, *Candida*, *Wickerhamomyces*, *Alternaria*, *Mucor*, *Epicoccum*, *Cutaneotrichosporon*, *Penicillium*, *Wallemia*, and *Sporobolomyces* showed a positive correlation (*p* < 0.05) with humidity, moisture, and acidity, and a negative correlation with oxygen content (*p* < 0.05). Environmental factors might have had opposite effects on the major bacterial and fungal species.

**Figure 4:**
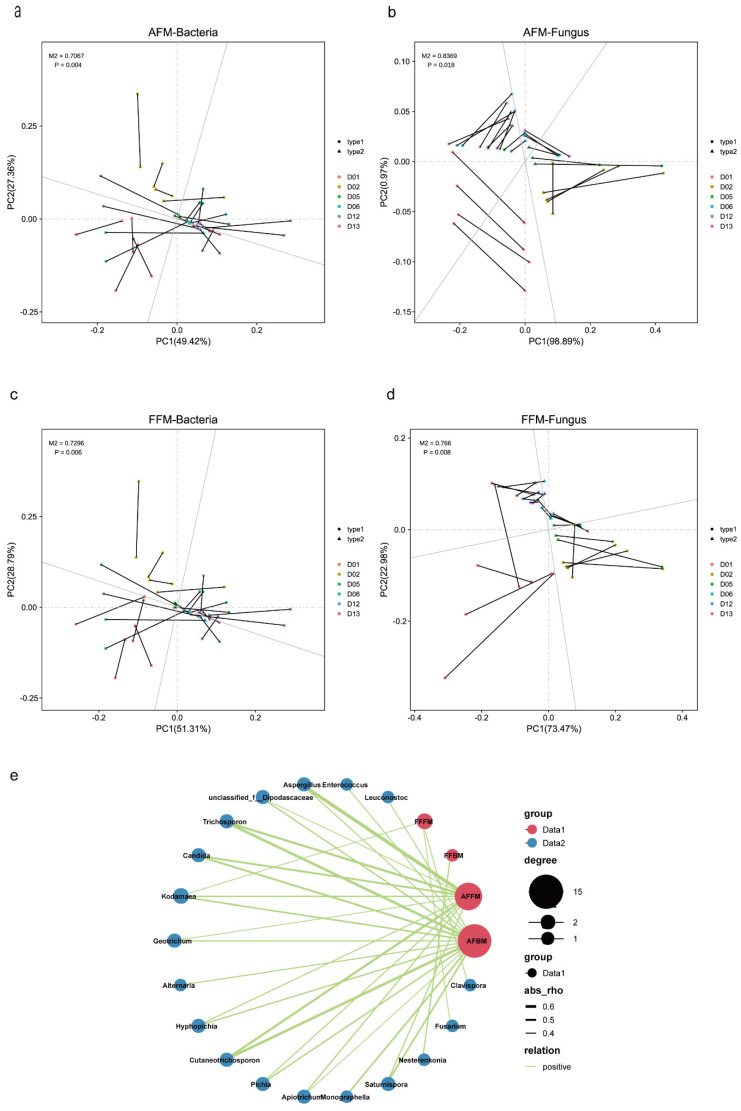
Effects of microbial interactions on functional microbial communities. Influence of bacterial and fungal communities on AFM (**a**,**b**) and FFM (**c**,**d**) by Procrustes analysis. Type1 represents bacterial or fungal communities, and Type2 represents the species. The length of the line is the residual value between the two, the shorter the line, the smaller the residual value. Network analysis of the correlation between functional microbial communities and other microbial communities (**e**). Red circles represent function microbial communities and blue circles represent species that are correlated with function microbial communities.

Procrustes analysis was performed to study microbial interactions. As shown in Figure 4a–d, the correlation between bacterial and fungal communities and AFM and FFM reached a significant level (*p* < 0.05), indicating that both bacterial and fungal communities could drive the succession of AFM and FFM. By co-occurrence network analysis in Figure 4e, (|R| > 0.4, *p* < 0.05), we found that AFM succession was affected by three bacterial genera and multiple yeast genera, and FFM succession was associated with one bacterial genus and three fungal genera. AFBM had higher co-occurrence with *Candida*, *Trichosporon*, and *Cutaneotrichosporon*, whereas AFFM succession was more significantly influenced by *Trichosporon*, *Cutaneotrichosporon*, and *Aspergillus*. *Nesterenkonia* had some influence on FFBM succession. *Kodamaea*, *Fusarium*, and *Clavispora* had an effect on FFFM succession. Therefore, microbial interactions could influence succession of FFM and AFM. The significant positive correlation between both communities and multiple microbes indicated that several microbes had a commensalism or synergism effect with both communities.

### 3.4. Potential Functions of the Two Microbial Communities

**Figure 5:**
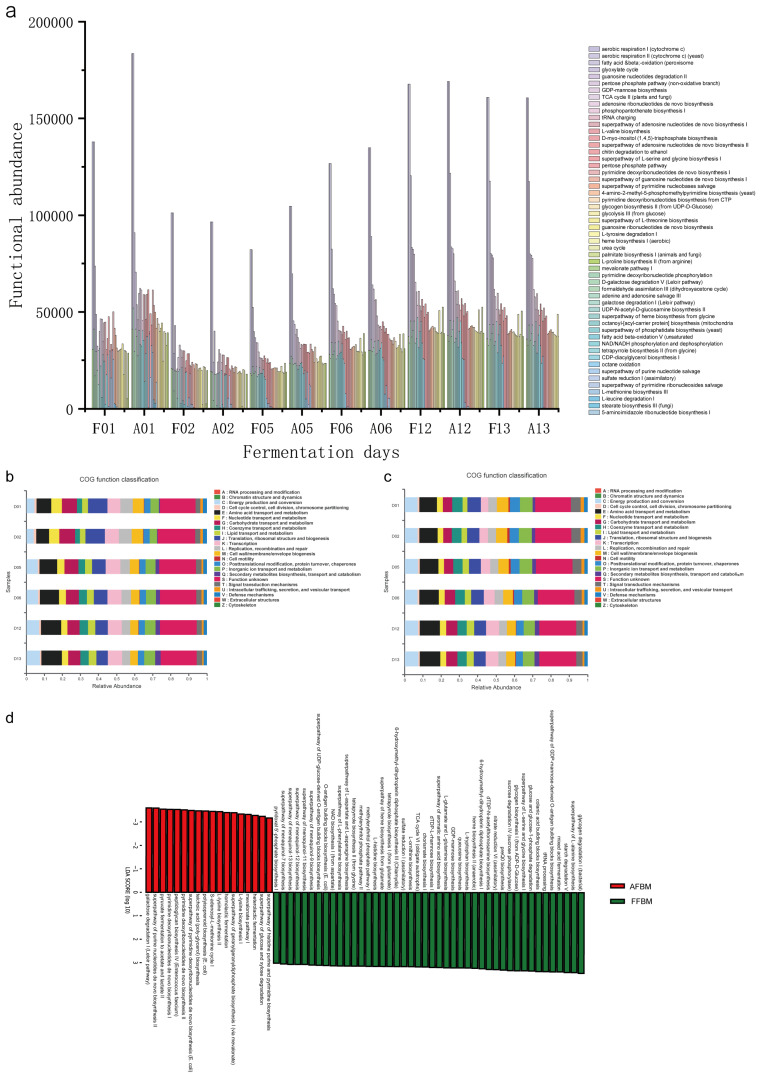
Functional abundance of FFFM and AFFM (**a**). In the horizontal coordinates, F represents FFFM, A represents AFFM, and function is expressed by the relative abundance of metabolic pathways. Clusters of orthologous groups functional classification of FFBM (**b**) and AFBM (**c**). Functional differences between FFBM and AFBM (**d**). Red represents the differential metabolic pathways in AFBM, green represents the differential metabolic pathways in FFBM. (Function prediction based on MetaCyc data).

To understand the functions of the associated microbial communities, functions were predicted using PICRUSt2. The MetaCyc pathway abundance data showed that 71 metabolic pathways were common to both FFFM and AFFM, except for one metabolic pathway that was unique to AFFM. Because of the low abundance of some metabolic pathways, only the main metabolic pathways were analyzed, i.e., the top 54 in abundance. The analysis of FFFM and AFFM functions (Figure 5a) revealed 6 metabolic pathways for carbohydrate biosynthesis/degradation; 7 metabolic pathways for precursor metabolites and energy production; 15 metabolic pathways for nucleotide metabolism (which might be involved in DNA/RNA biosynthesis); 7 amino acid metabolic pathways; 6 cofactor, carrier, and vitamin biosynthesis metabolic pathways; 7 fatty acid and lipid biosynthesis/degradation metabolic pathways; and 2 secondary metabolite biosynthesis metabolic pathways, indicating that the community was more active in all aspects of metabolic functions, especially in the utilization of raw materials for decomposition and autogenous reproduction. Enzymes encoding saccharification and ethanol fermentation-related enzymes might be associated with *Thermoascus* and *Thermomyces* [10], which were the absolute dominant fungi shared by FFFM and AFFM, which is consistent with our previous discussion that both fungi are important fermentation starter strains in *Baijiu* production.

The functional abundance of FFFM and AFFM (Figure 5a) was relatively consistent day to day. The metabolic pathways that were not significantly different between the two communities (LDA ≥ 3.0, *p* ≤ 0.05) were found using LEfSe on community functions, which showed that FFFM and AFFM functions were consistent. On further analysis, 13 metabolic pathways directly produced carbon dioxide and one metabolic pathway produced ethanol—superpathway II for adenosine nucleotide ab initio biosynthesis, heme biosynthesis I (aerobic), L-proline biosynthesis II (from arginine), TCA cycle II (fungi), mevalonate pathway I, L-valine biosynthesis, L-tyrosine degradation I, glycine superpathway for heme synthesis, stearic acid biosynthesis III (fungi), L-leucine degradation I, tetrapyrrole biosynthesis II (from glycine), octanoyl-[acyl carrier protein] biosynthesis (mitochondria), and chitin degradation to ethanol. Thus, carbon dioxide production pathways were more abundant than ethanol production pathways.

The clusters of orthologous groups (COG) functional abundance data indicated that the same 23 broad categories of functions were present in both FFBM and AFBM (Figure 5b,c). Analysis of FFBM and AFBM functions revealed that these functions were more abundant. It was more active in energy production and conversion, amino acid transport and metabolism, carbohydrate transport and metabolism, translation ribosome structure and biogenesis, transcription, and inorganic ion transport and metabolism. This indicated that the community was in a high rate of reproduction and had a high level of metabolic processes, which might help produce more intermediate metabolites that contribute to flavor formation. This also has some similarity to the function of high-temperature *Daqu* bacterial communities [39]. Further, LEfSe of community functions was performed to select differential metabolic pathways (LDA ≥ 3.0, *p* ≤ 0.05). The functions that showed significant differences in FFBM were coenzyme transport and metabolism, post-translational modification of protein turnover chaperones, cell motility, inorganic ion transport and metabolism, intracellular transport secretion and vesicular transport, and signal transduction mechanism (Figure S3), indicating that FFBM was active in product synthesis and transport-related metabolic functions. In AFBM, the functions that showed significant differences were carbohydrate transport and metabolism, acid transport and metabolism, transcription, recombination, and repair, indicating that AFBM was very active in raw material utilization and its own metabolic synthesis.

We analyzed the specific pathways to obtain detailed information. MetaCyc pathway abundance data showed 360 and 329 metabolic pathways in FFBM and AFBM, respectively, of which 326 metabolic pathways were common to both, 34 pathways were unique to FFBM, and only 3 pathways were unique to AFBM. LEfSe of metabolic pathway abundance differences showed that 62 metabolic pathways were significantly different (LDA ≥ 3.0, *p* ≤ 0.01), including 44 in FFBM and 18 in AFBM, a nearly three-fold difference (Figure 5d). FFBM had 10 amino acid biosynthesis pathways; 12 cofactor, carrier, and vitamin biosynthesis pathways; 12 carbohydrate metabolism pathways; 2 nucleic acid processing pathways; 2 precursor metabolism and energy production pathways; 3 secondary metabolite biosynthesis pathways; and 1 sulfur compound metabolism pathway. In addition, five of these metabolic pathways produce carbon dioxide—the superpathway of heme biosynthesis from glutamate, L-tryptophan biosynthesis, the superpathway of L-phenylalanine biosynthesis, heme biosynthesis II (anaerobic), and pyridoxal 5′-phosphate biosynthesis I. Thus, the community was mainly active at higher levels of primary and secondary metabolism, which was consistent with the differential analysis of COG functions. In AFBM, there were two amino acid biosynthesis pathways; two cofactor, carrier, and vitamin biosynthesis pathways; three carbohydrate metabolism pathways; two precursor metabolism pathways; two energy production pathways (both for lactic acid fermentation, of which iso-lactic acid fermentation produces ethanol); two secondary metabolite biosynthesis pathways; five nucleotide metabolism pathways; and two cell wall biosynthesis pathways. This coincided with the differential analysis of COG functions, where the nucleotide metabolism pathways confirmed that AFBM was active in its own transcription and recombination, and that lactate was produced in three carbohydrate metabolism pathways and two precursor metabolism and energy production pathways, indicating a high level of phosphorylation at the substrate level at this time.

In Figure 5d, some differential metabolic pathways of FFBM were connected before and after: (i) pentose phosphate pathway → branching acid biosynthesis → superpathway of aromatic amino acid biosynthesis (L-tryptophan biosynthesis, L-phenylalanine biosynthesis superpathway). The pentose phosphate pathway can produce D-erythrose-4-phosphate, which can be used as a starting substrate in branching acid biosynthesis, an important intermediate that can participate in the biosynthesis of many essential metabolites. (ii) L-glutamate and L-glutamine biosynthesis/L-serine and glycine biosynthesis superpathway I → tetrapyrrole biosynthesis I (from glutamate)/tetrapyrrole biosynthesis II (from glycine) → heme biosynthesis from glutamate/heme biosynthesis II (anaerobic). Heme is an iron-containing cofactor found in many essential proteins; in addition to its role in oxidative metabolism, it plays a molecular regulatory role in transcription, translation, protein targeting, protein stability, and cell differentiation. Metabolite delivery is one of the ways in which microbial interactions work [40], suggesting a strong mutual cooperation among microbes within FFBM.

**Figure 6:**
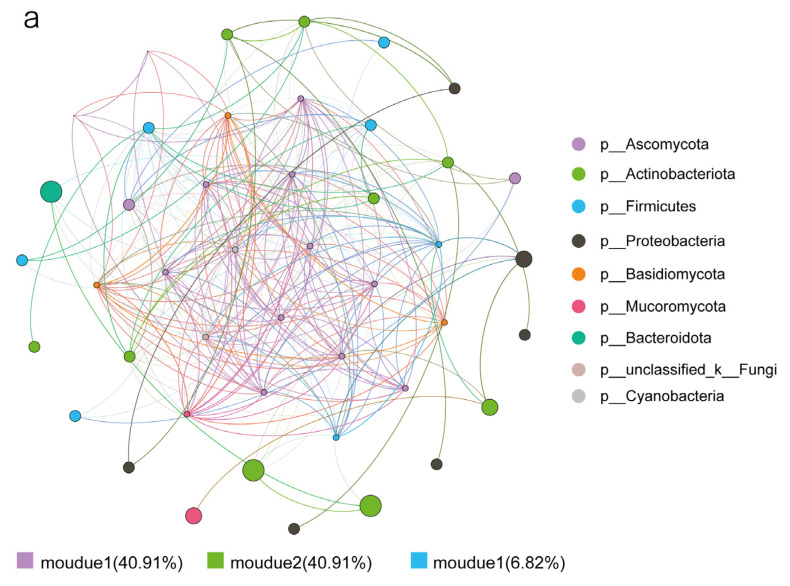

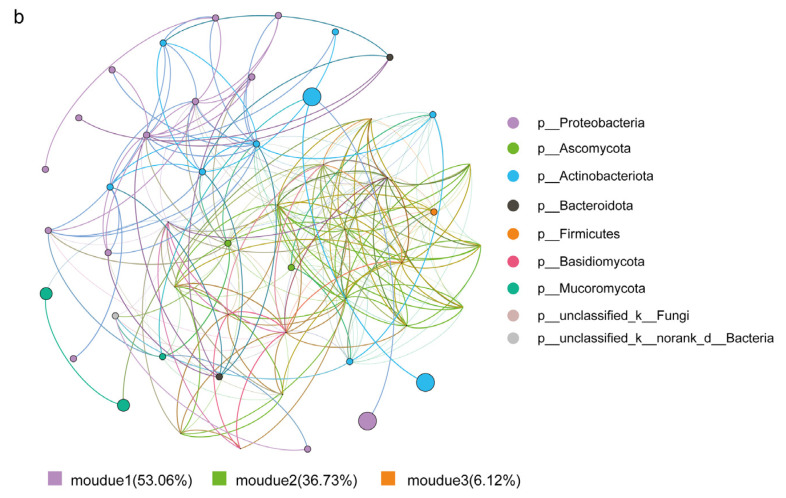
Co-occurrence network analysis of FFM (**a**) and AFM (**b**). Nodes are colored according to the phyla type, and nodes connected with the same color are of the same module. (r > 0.6, *p* < 0.05).

To understand the interactions within the functional microbial communities, genus-level co-occurrence network analysis was performed for FFM and AFM. As shown in Figure 6a,b, all nodes of the FFM and AFM co-occurrence networks were clustered into four modules. FFM mainly consisted of module 1 (40.92%) and module 2 (40.92%), and AFM mainly consisted of module 1 (53.06%) and module 2 (36.73%). However, the FFM network structure was more complex than the AFM network structure, and its modules 1 and 2 were strongly linked, indicating stronger FFM interactions and more active metabolic levels compared with the AFM network structure.

Based on the functions analysis, FFFM and AFFM functions converged in terms of kind and abundance. However, carbon dioxide production pathways were more than ethanol production pathways, indicating that the fermentation ability index was more widely representative of community activity. The number of differential functions was higher in FFBM than in AFBM, and the differential functions of FFBM indicated that the community was more advantageous for both substrate utilization and product synthesis. In summary, FFM was more widely functional and had a more active community than AFM.

## 4. Conclusions

In the present study, by analyzing the structure of microbial communities related to fermentation ability and alcohol production ability, we found that FFM and AFM were approximately the same at the phylum level but differed greatly at the genus level, and both occupied an important position in *Daqu* microbiota. Environmental factors significantly affected the structure the correlated communities. FFM and AFM were mainly affected by moisture, acidity, and humidity in the early stage of *Daqu* fermentation, and oxygen content was a critical factor for microbial succession in the middle stage of fermentation. Microbial interactions could drive the succession of both FFM and AFM, and multiple microbes had commensalism or synergism interactions in both communities. Function predictions revealed that FFFM and AFFM functions were relatively consistent, which was particularly active in the utilization of raw materials for decomposition and autogenous reproduction. FFBM was active in product synthesis and transport-related metabolic functions, while AFBM was very active in the utilization of raw materials and its own metabolic synthesis.

## Data Availability

The data presented in this study are available on request from the corresponding author.

## Supplementary Materials

The following supporting information can be downloaded at: https://www.mdpi.com/article/10.3390/foods11172602/s1, Figure S1: Sampling points of Daqu in the Qu-room; Figure S2: Variations in the fermentation ability and alcohol production ability. Red represents fermentation ability, blue represents alcohol production ability; Figure S3: Functional differences between FFBM and AFBM. Red represents the differential metabolic pathways in AFBM, green represents the differential metabolic pathways in FFBM. (Function prediction based on COG); Table S1: Functional community and environmental factor permutation test.
