# Supplementary material for: Structural Characteristics and Formation Mechanism of Microbiota Related to Fermentation Ability and Alcohol Production Ability in Nongxiang Daqu
Source: Foods. 2022 Aug 27;11(17):2602. doi: 10.3390/foods11172602 (PMC9455232; doi:10.3390/foods11172602)
Supplement: Supplementary file 1 [file foods-11-02602-s001.zip › foods-1854142-supplementary.pdf]

foods MDPI

Supplementary Information:

Table S1. Functional community and environmental factor permutation test.

permutation test	AFBM		AFFM		FFBM		FFFM	
	r2	Pr(>r)	r2	Pr(>r)	r2	Pr(>r)	r2	Pr(>r)
temporary	0.24	0.04	0.83	0.01	0.08	0.43	0.75	0.01
humidity	0.37	0.01	0.85	0.01	0.38	0.01	0.84	0.01
O2	0.43	0.01	0.50	0.01	0.43	0.01	0.54	0.01
Moisture	0.60	0.01	0.65	0.01	0.61	0.01	0.67	0.01
acidity	0.45	0.01	0.78	0.01	0.43	0.01	0.79	0.01

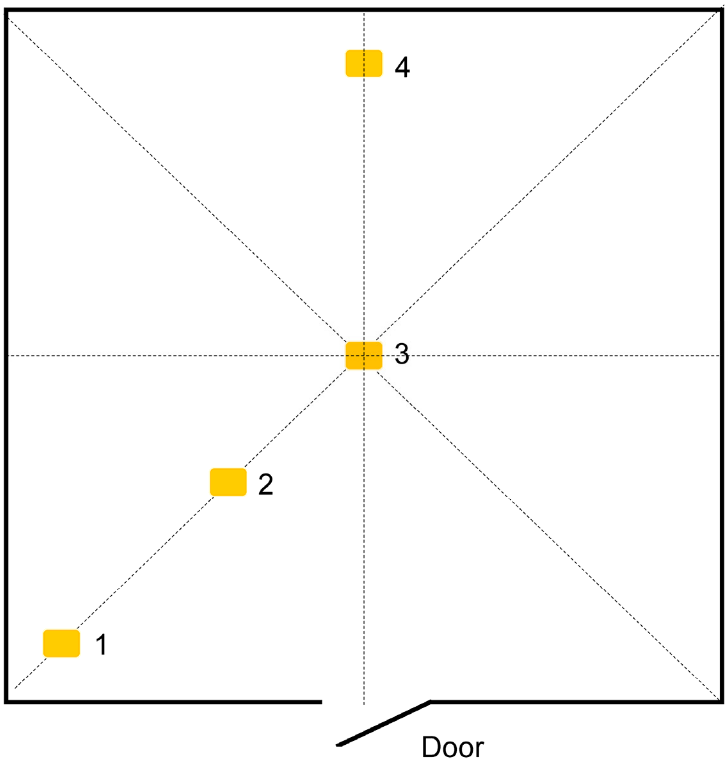

Figure S1. Sampling points of Daqu in the Qu-room.

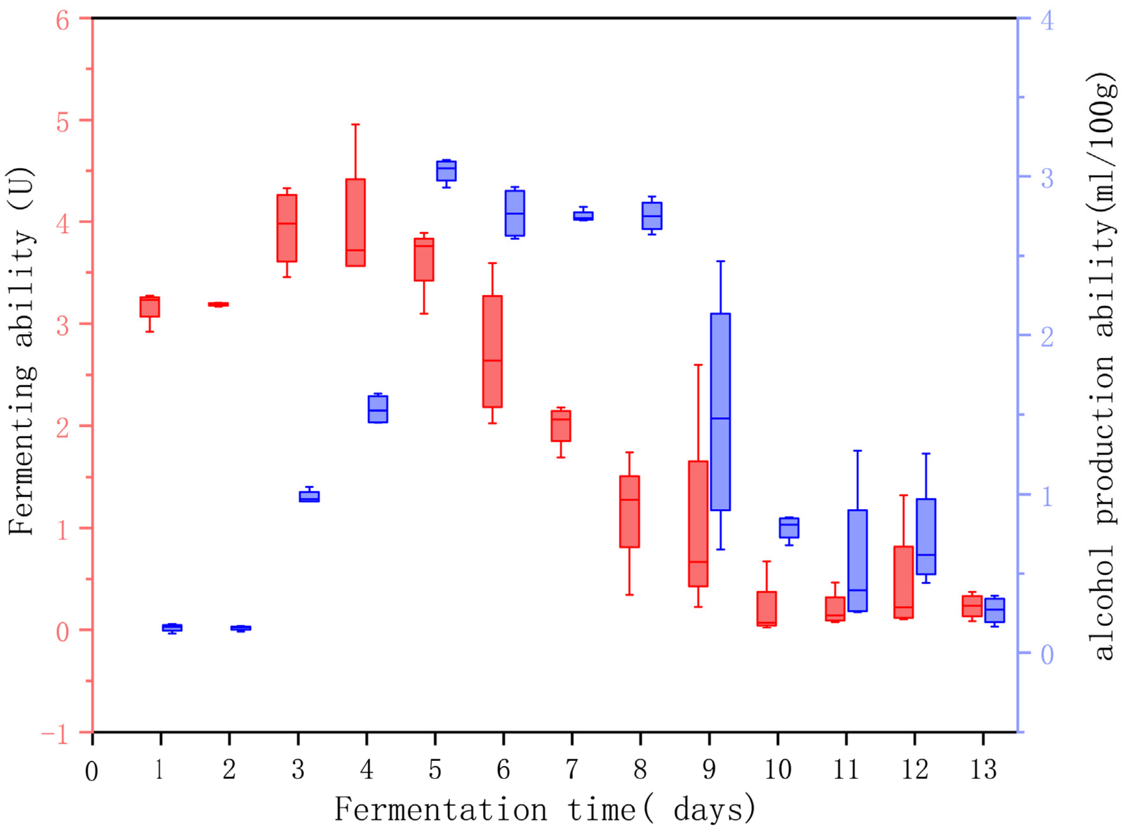

Figure S2. Variations in the fermentation ability and alcohol production ability. Red represents fermentation ability, blue represents alcohol production ability.

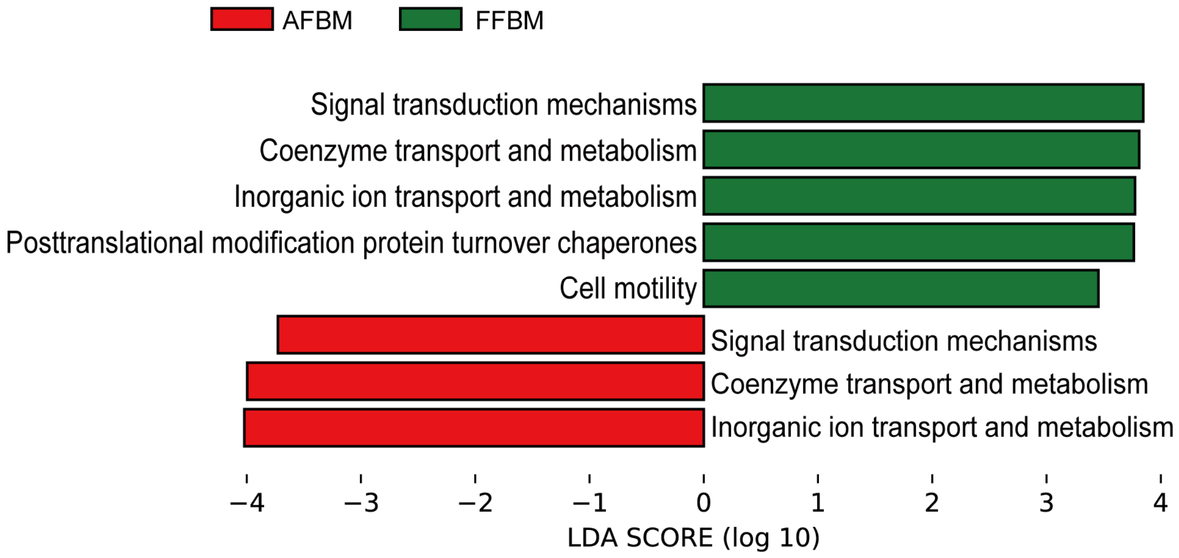

Figure S3. Functional differences between FFBM and AFBM. Red represents the differential metabolic pathways in AFBM, green represents the differential metabolic pathways in FFBM. (Function prediction based on COG).
